# Interaction between COX-1 and COX-2 increases susceptibility to ischemic stroke in a Chinese population

**DOI:** 10.1186/s12883-019-1505-1

**Published:** 2019-11-17

**Authors:** Lei Zhao, Jinghuan Fang, Muke Zhou, Jie Zhou, Lihua Yu, Ning Chen, Li He

**Affiliations:** Department of Neurology, West China Hospital of Sichuan University, No.37, Guoxue Road, Chengdu, Sichuan 610041 People’s Republic of China

**Keywords:** Ischemic stroke, *COX-1*, *COX-2*, Single nucleotide polymorphism, Gene-gene, Interaction

## Abstract

**Background:**

Mutations of cyclooxygenase gene (COX gene) may increase the susceptibility of ischemic stroke. We investigated five variants (rs5788, rs1330344, rs3842788, rs20417, and rs689466) of two COX genes in order to explaining the association between these polymorphisms and we also investigated the association between these variants and ischemic stroke risk to determine whether gene–gene interaction between these genes increases the susceptibility of ischemic stroke or its subtypes.

**Methods:**

A total of 1981 study subjects (1078 cases and 903 control subjects) were recruited. The interaction of multiple factors was investigated using Multifactor Dimensionality Reduction. The additive effect of single nucleotide polymorphisms on ischemic stroke or its subtypes were analyzed by multiple factor logistic regression.

**Results:**

At COX-1(rs1330344), AA genotype carriers had a lower susceptibility of ischemic stroke (*OR = 0.657, 95%CI = 0.437–0.988, P = 0.044*), and A allele carriers had a lower susceptibility of ischemic stroke (*OR = 0.812, 95%CI = 0.657–0.978, P = 0.029*). At COX-1(rs3842788), AA genotype carriers had a higher susceptibility of ischemic stroke (*OR = 5.203, 95% CI = 1.519–5.159, P = 0.016)*. At COX-2 (rs689466), AA genotype carriers had a higher susceptibility of large-artery atherosclerosis (*OR = 1.404, 95% CI = 1.019–1.934, P = 0.038*). COX-1(rs1330344, rs3842788) and COX-2 rs689466 interacted in SVO, but had no additive effect with ischemic stroke and other subtypes.

**Conclusions:**

At rs1330344, AA genotype may reduce the susceptibility of ischemic stroke. At rs3842788, AA genotype may increase the susceptibility of ischemic stroke. At rs689466, AA genotype may increase the susceptibility of large-artery atherosclerosis (LAA). COX − 1(rs1330344, rs3842788) and COX-2 rs689466 interacted in small vessel occlusion (SVO), but had no additive effect with ischemic stroke and other subtypes.

## Background

Stroke is a common cerebrovascular disease, which is the second cause of disability and mortality worldwide. The most common type of stroke is ischemic stroke, which accounts for approximately 80% of all stroke cases [1, 2]. As a complex disease, ischemic stroke was characterized by several etiological factors and likely affected by various genes, environments, lifestyles, and other causes; which could produce complex high-order interactions to this disease [3–6].

Researchers had found that lipid metabolism, blood coagulation, fibrinolysis system and inflammatory factors played a very important role in the pathogenesis of ischemic stroke [5]. On one hand, the mutation of some gene involved in the inflammatory response can cause the internal environment disturbance which might lead to the occurrence and development process of atherosclerosis. On the other hand, ultimately unstable or ruptured atherosclerotic plaque will cause the occurrence of acute cerebrovascular diseases. Therefore, Atherosclerosis is the main pathophysiological basis of both cardiovascular and cerebrovascular diseases. Atherosclerosis is a kind of multifactorial disease, involving many factors such as gene, environment, metabolic interaction at the same time and also a kind of participation by a variety of inflammatory cytokines and cell proliferation of chronic inflammation [7]. Furthermore, the adhesion, aggregation and release of platelet is another important part of the thrombosis. The platelet adhesive phenomenon (platelet aggregation) is closely related to thrombosis. In recent years, many scholars have found that the metabolic product of arachidonic acid (AA)plays an important role in the formation of atherosclerosis and thrombosis and is closely related with the incidence of ischemic stroke. Meanwhile AA metabolic disorders affects the stability of vascular endothelium, vasomotor function and platelet aggregation, which lead to the occurrence of vessel diseases [8, 9].

Genome-wide association studies (GWAS)have verified various ischemic stroke-associated inflammatory factor genes, including cyclooxygenase or prostaglandin oxidase (COX) [10–17]. COX is a bifunctional enzyme with oxidase and catalase activities. It is also a key enzyme in the arachidonic acid metabolism of prostaglandins (PGs) and thromboxane A2 (TXA2). Prostaglandin E2(PGE2) is involved in inflammation, and TXA2 is implicated in platelet aggregation and vasoconstriction [16]. COX gene is closely related to ischemic stroke, elucidating its genetic mechanism has a great significance in exploring the risk prediction of ischemic stroke and developing new targets for drug treatment. There are two forms of COX in the human body currently: COX-1 and COX-2. One is the structural type COX-1, which is stably expressed in normal cells, catalyzes the formation of PGs to maintain normal physiological function, regulates platelet functional activity and coagulation function. And the other is induced type COX-2, which is only highly expressed when the cells affected by various factors to participates in physiological and pathological processes of inflammation or tumor such as oncogenes, cytokines, growth factor, endotoxin, carcinogen, catalyzes prostaglandin E 2 (PGE2) [18]. Based on this physiological role, we assumed that the mutation of COX gene may be associated with the susceptibility of ischemic stroke. Maree et al. [19] has demonstrated that COX-1 gene mutation can change the platelet aggregation rate and affect thrombus formation, therefore it would cause thrombotic diseases such as ischemic stroke by regulating arachidonic acid (AA) metabolic processes. GWAS and other related studies have revealed that multiple SNPs of a COX-2 gene can influence inflammatory processes in the human body by regulating the expression of its products, such as PGs increasing the formation of plaque [17]. And there was a study showed that COX-2 is an ischemic stroke-susceptible gene in African–American and European populations [20].

However, there are some controversial researches of COX-1 and COX-2. There is no specific locus associated with ischemic stroke, and the results varied from population to population [21–26]. It maybe because it is hard to use the statistical significance of single SNP studies on ischemic stroke to explain the association between SNPs in the same gene. Therefore, it is very important for us to consider the interacting effects of multiple SNPs on ischemic stroke. So we conducted a large-sample case-control study to investigate the association of five variants (rs5788, rs1330344, rs3842788, rs20417, and rs689466) of COX-1 and COX-2 genes with ischemic stroke and to investigate the pattern of genetic variation within COX and to explore whether gene–gene interactions between these genes increase the susceptibility of ischemic stroke or its subtypes.

## Methods

### Samples

Genomic DNA was isolated from morning fasting peripheral venous blood of 1078 cases of patients with diagnosed of ischemic stroke (ischemic stroke group) and 903 cases of non-ischemic stroke subjects (control group) from the Han racial/ethnic groups in Mainland China. All the patients came from the same region of the Han population and were matched in terms by gender and age. Patients’ information was obtained from the stroke database of the Neurology Department of West China Hospital.

The inclusion criteria were as follows: 1) age ≥ 40 years old; 2) the diagnose of ischemic stroke was confirmed by brain magnetic resonance imaging according to WHO diagnostic criteria [27]. Exclusion criteria was as follows: 1) hemorrhagic stroke, or recurrent ischemic stroke; 2) other etiologies of ischemic stroke: stroke of other determined etiology (SOD), or stroke of undetermined etiology (SUD); 3) cerebrovascular malformation or brain tumor. In this study, ischemic stroke group were divided into three subtypes: large-artery atherosclerosis (LAA), cardioembolic stroke (CES), and small vessel occlusion (SVO) according to TOAST typing [28]. We selected the patients without stroke history and none cerebral infarction lesions in CT/MRI into control group. The purpose of the study was described in detail, and informed consents were obtained from all the participants. This study was approved by the Ethics Review Committee of West China hospital, Sichuan University.

### Demographic data and characteristics of participants

The following demographic data and relevant characteristics were obtained: hypertension (SBP ≥ 140 mmHg/DBP ≥ 90 mmHg) or medication intake, diabetes (random blood glucose ≥7.0 mmol/L) or medication intake, coronary heart disease, hyperlipidemia (cholesterol, TC ≥ 5.62 mmol/L/, or triglyceride, TG ≥ 1.92 mmol/L) or medication intake, smoking (≥ 10 cigarettes per day, smoking age > 1 years, or duration of smoking cessation ≤3 years), [29] and drinking (> 50 ml/d, > 1 year). Other data were also collected from patients’ medical records.

### Genotype determination

Genomic DNA was isolated with an AxyPrepTM blood genomic DNA maxiprep kit (Axygen Biosciences, USA). Five SNPs of *COX-1* and *COX-2* were retrieved from the NCBI database (http://www.ncbi.nlm.nih.gov/SNP). Five SNPs selected for our study (based on potential functional significance and minor allele frequency of > 0.10)-*COX-1-644C/A* (*rs5788*), *COX-1-1676G/A* (*rs1330344*), *COX-1-128G/A* (*rs3842788*), *COX-2-765G*/*C* (*rs20417*), and *COX-2-1195G/A* (*rs689466*) [30]. Genotyping was performed in a blinded manner by K-Biosciences (Herts, UK) (http://www.kbioscience.co.uk/) using a competitive allele-specific PCR system (KASPar).

### Statistical analysis

Three common genetic models are used in related studies: dominant, recessive, and additive model. In this study, each SNP contained the major allele A and minor allele B. AB+BB versus AA was calculated by the dominant model, BB versus AB+AA was determined by the recessive model, and AB versus AA or BB versus AA was examined by the additive model. The three models for each site were statistically analyzed by SPSS 19.0 (SPSS Inc., Chicago, IL, USA). The frequencies of each allele and genotype were calculated by performing χ^2^ test and described as mean ± SD. The Hardy–Weinberg equilibrium of the five polymorphic distributions of the patients and controls was evaluated through a chi-square goodness-of-fit test. The correlation of the SNPs and their cumulative effects on ischemic stroke were examined through multiple factor logistic regression after adjusted related risk factors, such as gender, age, smoking, and hypertension. The interaction of multiple factors was investigated using MDR version 3.0.2 (http://sourceforge.net/projects/mdr/). Odds ratios were calculated with 95% confidence intervals. *P* < 0.05 was considered statistically significant.

## Results

### Study population and baseline characteristics

There were in total 1981 subjects, including 1078 cases of ischemic stroke patients and 903 cases of control group. The clinical characteristics of the patients and controls were listed in Table 1. The proportion was higher of hypertension, diabetes, hyperlipidemia, and coronary heart disease, smoking in patient group, and also higher fibrinogen level, lower high-density lipoprotein cholesterol (HDL-C) level and lower blood platelet (PLT) level. There were significant differences of the proportion of men in our study between the case group and the control group. LDL-C level between two groups had no significant difference. These risk factors such as sex, hypertension, diabetes, smoking, alcohol consumption, triglyceride level, platelet level and fibrinogen level were associated with the three subtypes— LAA, CES and SVO groups of ischemic stroke, and the distribution of the three groups was also statistical significantly different.

**Table 1:** Baseline Characteristics for INVEST-GENES Participants

Characteristic	Control group(*n* = 903)	*IS* group (*n* = 1078)	*P* value	
Age(years)	57.0 ± 14.9	64.4 ± 12.3	0.486	
**Sex(male%)**	474(52.5)	677(62.8)	**< 10**^**− 3**^	
**Hypertension (%)**	154(17.1)	729(67.6)	**< 10**^**− 3**^	
**Diabetes (%)**	59(6.5)	361(33.5)	**< 10**^**−3**^	
**Hyperlipidemia (%)**	90(10.0)	177(16.4)	**< 10**^**−3**^	
**Coronary heart disease (%)**	10(1.1)	115(10.7)	**< 10**^**−3**^	
**Smoking (%)**	244(27.0)	369 (34.2)	**0.001**	
Alcohol consumption(%)	175(19.6)	235(21.9)	0.214	
TG(mmol/L)	1.50 ± 1.13	2.27 ± 24.2	0.342	
TC(mmol/L)	4.36 ± 1.03	4.27 ± 2.73	0.311	
**HDL-C(mmol/L)**	1.36 ± 0.43	1.25 ± 0.50	**< 10**^**−3**^	
LDL-C(mmol/L)	2.73 ± 7.05	2.47 ± 0.96	0.230	
**Fibrinogen levels (mmol/L)**	2.20 ± 1.59	3.16 ± 1.00	**< 10**^**−3**^	
**Plt(10^9/L)**	178.5 ± 72.1	169.0 ± 66.70	**0.021**	
Characteristic	*LAA* group (*n* = 405)	*CES* group(*n* = 136)	*SVO group(n = 537)*	*P value*
Age(years)	63.2 ± 12.6	64.5 ± 12.9	64.9 ± 11.9	0.079
**Sex(male%)**	274(67.7)	62(45.6)	341(63.5)	**< 10**^**−3**^
**Hypertension (%)**	268(66.2)	65(47.8)	396(67.6)	**< 10**^**−3**^
**Diabetes (%)**	132(32.6)	24(17.6)	205(38.2)	**< 10**^**−3**^
Hyperlipidemia (%)	68(16.8)	13(9.6)	96(17.9)	0.063
Coronary heart disease **(%)**	45(11.1)	18(13.2)	52(9.7)	0.457
**Smoking (%)**	162(40.0)	25(18.4)	182(33.9)	**< 10**^**−3**^
**Alcohol consumption(%)**	102 (25.2)	17(12.6)	116(21.7)	**0.009**
**TG(mmol/L)**	1.65 ± 1.22	1.26 ± 0.66	2.52 ± 27.79	**0.042**
TC(mmol/L)	4.51 ± 4.88	4.00 ± 1.58	4.21 ± 1.15	0.367
HDL-C(mmol/L)	1.24 ± 0.68	1.30 ± 0.43	1.24 ± 0.40	0.926
LDL-C(mmol/L)	2.44 ± 0.94	2.64 ± 3.87	2.49 ± 0.99	0.084
**Fibrinogen levels (mmol/L)**	3.22 ± 0.97	2.98 ± 1.04	3.16 ± 1.00	**0.012**
**Plt(10^9/L)**	171.2 ± 63.5	151.5 ± 61.0	171.0 ± 67.2	**0.003**

count data:mean ± SD, measurement data:(%)

*P* < 0.05 in bold

### Genotype and allele frequency distributions of SNPs in ischemic stroke and controls(Table 2)

At *COX-1* (*rs5788*) and*COX-2* (*rs20417*), no significant differences were observed in genotype and allele frequencies between the ischemic stroke group including the three subtypes (LAA, CES, SVO) and the control group, even after we using multivariate analysis by adjusting for age, gender and related risk factors.

**Table 2:** Genotype and allele frequency distributions of SNPs in IS and controls

	Control group (%)	*IS* group			*LAA* group		*CES* group		*SVO* group	
		*n*(%)	*P* value	OR(95%CI)	*P* value	OR(95%CI)	*P* value	OR(95% CI)	*P* value	OR(95% CI)
rs5788										
Genotype										
CC	810 (89.7)	974 (90.4)	–	Reference	–	Reference	–	Reference	–	Reference
CA	89 (9.9)	102 (9.5)	0.384	2.634 (0.297–23.332)	0.657	0.894 (0.545–1.466)	0.465	1.254(0.684–2.298)	0.433	0.824 (0.507–1.338)
AA	4 (0.4)	2 (0.2)	0.426	2.450 (0.270–22.255)	0.818	0.742 (0.058–9.471)	NA	–	0.539	0.380 (0.017–8.288)
CA + AA vs CC			0.600	0.907 (0.629–1.307)	0.634	0.888 (0.546–1.446)	0.525	1.216 (0.665–2.224)	0.383	0.808 (0.500–1.305)
AA vs CC + CA			0.388	0.382 (0.043–3.387)	0.825	0.750 (0.059–9.572)	NA	–	0.546	0.386 (0.018–8.454)
Allele										
C	1709 (94.6)	2050 (95.1)	–	Reference	–	Reference	–	Reference	–	Reference
A	97 (5.4)	106 (4.9)	0.498	0.884 (0.618–1.263)	0.510	0.851 (0.528–1.374)	0.586	1.173 (0.660–2.085)	0.413	0.824 (0.519–1.309)
rs1330344										
Genotype										
GG	126 (14.0)	177 (16.4)	–	Reference	–	Reference	–	Reference	–	Reference
GA	456 (50.5)	510 (47.3)	0.350	0.827 (0.555–1.232)	0.426	0.815 (0.402–1.350)	0.503	0.809 (0.436–1.503)	0.207	0.739 (0.463–1.182)
**AA**	321 (35.5)	391 (36.3)	**0.044**	0.657 (0.437–0.988)	0.108	0.652 (0.387–1.099)	**0.043**	0.506 (0.262–0.978)	**0.048**	0.616 (0.382–0.995)
GA + AA vs GG			0.133	0.794 (0.587–1.073)	0.239	0.781 (0.517–1.179)	0.178	0.699 (0.415–1.177)	0.050	0.690 (0.475–1.000)
AA vs GG + GA			0.588	0.939 (0.748–1.179)	0.623	0.927 (0.685–1.254)	0.132	0.723 (0.474–1.102)	0.698	0.944 (0.708–1.260)
Allele										
G	708 (39.2)	864 (40.1)	–	Reference	–	Reference	–	Reference	–	Reference
**A**	1098 (60.8)	1292 (59.9)	**0.029**	0.812 (0.675–0.978)	**0.034**	0.778 (0.618–0.981)	**0.023**	0.702 (0.518–0.951)	0.097	0.829(0.665–1.035)
rs3842788										
Genotype										
GG	795 (88.0)	890 (82.6)	–	Reference	–	Reference	–	Reference	–	Reference
GA	97 (10.7)	120 (11.1)	0.337	0.821 (0.549–1.228)	0.122	0.652 (0.379–1.122)	0.861	0.944 (0.495–1.801)	0.591	0.876 (0.540–1.420)
AA	11 (1.3)	68 (6.3)	0.165	5.127 (0.511–5.428)	0.128	7.858 (0.552–111.969)	0.082	9.758 (0.748–12.273)	0.326	4.586 (0.219–5.902)
GA + AA vs GG			0.487	0.868 (0.583–1.293)	0.206	0.711 (0.419–1.207)	0.882	1.048 (0.562–1.955)	0.692	0.908 (0.563–1.465)
**AA vs GG + GA**			**0.016**	5.203 (1.519–5.159)	0.121	8.167 (0.573–16.414)	**0.041**	9.821 (2.754–12.832)	**0.025**	4.603 (1.220–5.201)
Allele										
G	1687 (93.4)	1900 (88.1)	–	Reference	–	Reference	–	Reference	–	Reference
A	119 (6.6)	256 (11.9)	0.699	0.928 (0.635–1.355)	0.354	0.789 (0.479–1.302)	0.620	1.157 (0.650–2.057)	0.817	0.947 (0.600–1.496)
rs20417										
Genotype										
GG	812 (89.9)	977 (90.6)	–	Reference	–	Reference	–	Reference	–	Reference
GC	87 (9.6)	97 (9.0)	0.680	1.080 (0.748–1.561)	0.424	1.223 (0.746–2.006)	0.812	1.084 (0.556–2.114)	0.423	1.211 (0.758–1.935)
CC	4 (0.4)	4 (0.4)	0.341	421 (0.071–2.500)	0.528	0.443 (0.035–5.560)	0.479	0.335 (0.016–6.937)	0.528	0.499 (0.058–4.315)
GC + CC vs GG			0.820	1.043 (0.726–1.498)	0.513	1.177 (0.723–1.915)	0.953	1.020 (0.528–1.970)	0.513	1.166 (0.736–1.848)
CC vs GG + GC			0.378	0.418 (0.070–2.482)	0.519	0.436 (0.035–5.460)	0.477	0.334 (0.016–6.899)	0.518	0.492 (0.057–4.239)
Allele										
G	1711 (94.7)	2051 (95.1)	–	Reference	–	Reference	–	Reference	–	Reference
C	95 (5.3)	105 (4.9)	0.952	0.987 (0.655–1.489)	0.630	1.137 (0.675–1.914)	0.963	0.984 (0.504–1.924)	0.646	1.124 (0.684–1.847)
rs689466										
Genotype										
GG	189 (20.9)	241 (22.4)	–	Reference	–	Reference	–	Reference	–	Reference
GA	472 (52.3)	519 (48.1)	0.408	0.888 (0.671–1.176)	0.435	0.861 (0.592–1.253)	0.676	1.116 (0.666–1.872)	0.230	0.805 0.565–1.147)
AA	242 (26.8)	318 (29.5)	0.535	1.104 (0.807–1.511)	0.262	1.266 (0.838–1.910)	0.871	1.050 (0.581–1.897)	0.883	1.030 (0.692–1.533)
GA + AA vs GG			0.758	0.959 (0.735–1.251)	0.960	0.991 (0.697–1.409)	0.791	1.095 (0.667–1.797)	0.438	0.876 (0.627–1.224)
**AA vs GG + GA**			0.140	1.201 (0.941–1.531)	**0.038**	1.404 (1.019–1.934)	0.890	0.969 (0.617–1.520)	0.251	1.200 (0.879–1.638)
Allele										
G	850 (47.1)	1001 (46.4)	–	Reference	–	Reference	–	Reference	–	Reference
A	956 (52.9)	1155 (53.6)	0.969	0.996 (0.829–1.198)	0.541	1.076 (0.851–1.360)	0.799	0.962 (0.712–1.300)	0.678	0.954 (0.764–1.192)

Using multivariate analysis after adjusting for age, gender and traditional risk factors

*P* < 0.05 in bold

*NA* Not available

Reference: means allele homozygote as a reference

At COX-1(rs1330344), compared to the GG genotype, the AA genotype carriers had a lower susceptibility of ischemic stroke, and this difference remained significant in multivariate analysis by adjusting for age, gender and traditional risk factors *(OR = 0.657, 95% CI = 0.437–0.988, P = 0.044).* And the AA genotype carriers also had a lower susceptibility of CES or SVO *(OR = 0.506, 95% CI = 0.262–0.978, P = 0.043; OR = 0.616, 95% CI = 0.382–0.995, P = 0.048).* Meanwhile, compared to the G allele, the A allele carriers had a lower susceptibility of ischemic stroke, and this difference remained significant in multivariate analysis by adjusting for age, gender and traditional risk factors *(OR = 0.812, 95% CI = 0.675–0.978, P = 0.029).* And the A allele carriers also had a lower susceptibility of LAA or CES *(OR = 0.778, 95% CI = 0.618–0.981, P = 0.034; OR = 0.702, 95% CI = 0.518–0.951, P = 0.023).*

At *COX-1*(*rs3842788*), compared to the G allele carriers genotype (GG + GA), the AA genotype carriers had a higher susceptibility of ischemic stroke, this difference remained significant in multivariate analysis by adjusting for age, gender and traditional risk factors *(OR = 5.203, 95% CI = 1.519–5.159, P = 0.016)*. And the AA genotype carriers also had a higher susceptibility of CES or SVO *(OR = 9.821, 95% CI = 2.754–12.832, P = 0.041; OR = 4.603, 95% CI = 1.220–5.201, P = 0.025).*

At *COX-2*(*rs689466*), compared to the G allele carriers genotype (GG + GA), the AA genotype carriers had a higher risk of LAA, this difference remained significant in multivariate analysis by adjusting for age, gender and traditional risk factors (*OR = 1.404, 95% CI = 1.019–1.934, P = 0.038*).

### Genotype and allele frequency distributions of SNPs in hypertension/diabetes/smoking and their controls**(**Table 3**)**

We respectively divided all participants into high risk factor group (hypertension /diabetes/smoking groups) and control group.

**Table 3:** Genotype and allele frequency distributions of SNPs in hypertension/diabetes/smoking and their controls

	Hypertension			Diabetes			Smoking		
	control = 1098(%)	*n* = 883(%)	*P* value	control = 1561(%)	*n* = 420(%)	*P* value	control = 1368(%)	*n* = 613(%)	*P* value
rs5788									
CC	991(90.3)	793(89.8)	–	1399(89.6)	385(91.7)	–	1242(90.8)	542(88.4)	–
CA	103(9.4)	88(10.0)	0.915	156(10.0)	35(8.3)	0.259	123(9.0)	68(11.1)	0.050
AA	4(0.4)	2(0.2)	0.947	6(0.4)	0	NA	3(0.2)	3(0.5)	0.269
CA + AA vs CC	–	–	0.338	–	–	0.195	–	–	**0.034**
AA vs CC + CA	–		0.930	–	–	NA	–	–	0.286
C	2085(94.9)	1674(94.8)	–	2954(94.6)	805(95.8)	–	2607(95.3)	1152(94.0)	–
A	111(5.1)	92(5.2)	0.502	168(5.4)	35(4.2)	0.084	129(4.7)	74(6.0)	0.081
rs1330344									
GG	168(15.3)	135(15.3)	–	229(14.7)	74(17.6)	–	218(15.9)	85(13.9)	–
GA	549(50.0)	417(47.2)	0.188	790(50.6)	176(41.9)	**0.047**	654(47.8)	312(50.9)	0.102
AA	381(34.7)	331(37.5)	0.121	542(34.7)	170(40.5)	0.887	496(36.3)	216(35.2)	0.133
GA + AA vs GG	–	–	0.128	–	–	0.270	–	–	0.092
AA vs GG + GA	–	–	0.388	–	–	**0.025**	–	–	0.662
G	885(40.3)	687(38.9)	–	1248(40.0)	324(38.6)	–	1090(39.8)	482(39.3)	–
A	1311(59.7)	1079(61.1)	0.284	1874(60.0)	516(61.4)	0.470	1646(60.2)	744(60.7)	0.649
rs3842788									
GG	978(89.1)	779(88.2)	–	1378(88.3)	379(90.2)	–	1198(87.6)	559(91.2)	–
GA	118(10.7)	97(11.0)	0.381	176(11.3)	39(9.3)	0.337	163(11.9)	52(8.5)	**0.030**
AA	2(0.2)	7(0.8)	0.286	7(0.4)	2(0.5)	0.474	7(0.5)	2(0.3)	0.506
GA + AA vs GG	–	–	0.284	–	–	0.274	–	–	**0.025**
AA vs GG + GA	–	–	0.294	–	–	0.490	–	–	0.535
G	2074(94.4)	1655(93.7)	–	2932(93.9)	797(94.9)	–	2559(93.50	1170(95.4)	–
A	122(5.6)	111(6.3)	0.173	190(6.1)	43(5.1)	0.197	177(6.5)	56(4.6)	**0.023**
rs20417									
GG	988(90.0)	801(90.7)	–	1399(89.6)	390(92.9)	–	1236(90.4)	553(90.2)	–
GC	107(9.7)	77(8.7)	0.868	157(10.1)	27(6.4)	**0.036**	124(9.1)	60(9.8)	0.733
CC	3(0.3)	5(0.6)	0.249	5(0.3)	3(0.7)	0.306	8(0.6)	0	NA
GC + CC vs GG	–	–	0.942	–	–	0.067	–	–	0.955
CC vs GG + GC	–	–	0.248	–	–	0.285	–	–	NA
G	2083(94.9)	1679(95.1)	–	2955(94.7)	807(96.1)	–	2596(94.9)	1166(95.1)	–
C	113(5.1)	87(4.9)	0.762	167(5.3)	33(3.9)	0.093	140(5.1)	60(4.9)	0.881
rs689466									
GG	241(21.9)	189(21.4)	–	324(20.8)	106(25.2)	–	297(21.7)	133(21.7)	–
GA	560(51.0)	431(48.8)	0.374	786(50.4)	205(48.8)	0.229	692(50.6)	299(48.8)	0.792
AA	297(27.0)	263(29.8)	0.063	451(28.9)	109(26.0)	**0.036**	379(27.7)	181(29.5)	0.745
GA + AA vs GG	–	–	0.164	–	–	0.085	–	–	0.750
AA vs GG + GA	–	–	0.094	–	–	0.087	–	–	0.835
G	1042(47.4)	809(45.8)	–	1434(45.9)	417(49.6)	–	1286(47.0)	565(46.1)	–
A	1154(52.6)	957(54.2)	0.063	1688(54.1)	423(50.4)	**0.025**	1450(53.0)	661(53.9)	0.317

Using multivariate analysis after adjusting for age, gender and traditional risk factors

*P* < 0.05 in bold

As for hypertension group, at all the five SNPs, no significant differences were observed in genotype and allele frequencies between the hypertension group and control group. After we using multivariate analysis by adjusting for age, gender and related risk factors, the result remains negative.

As for diabetes group, (1) At COX-1(rs1330344), compared to the GG genotype, the GA genotype carriers had a lower susceptibility of diabetes, and this difference remained significant in multivariate analysis by adjusting for age, gender and traditional risk factors*(OR = 0.711, 95% CI = 0.507–0.996, P = 0.047)*, and compared to the G allele carriers genotype (GG + GA), the AA genotype carriers had a higher susceptibility of diabetes, and this difference remained significant in multivariate analysis by adjusting for age, gender and traditional risk factors*(OR = 1.319, 95% CI = 1.036–1.680, P = 0.025).*(2)At COX-2(rs20417), compared to the GG genotype, the GC genotype carriers had a lower susceptibility of diabetes, and this difference remained significant in multivariate analysis by adjusting for age, gender and traditional risk factors*(OR = 0.616, 95% CI = 0.391–0.969, P = 0.036).*(3) At COX-2(rs689466), compared to the GG genotype, the AA genotype carriers had a lower susceptibility of diabetes, and this difference remained significant in multivariate analysis by adjusting for age, gender and traditional risk factors*(OR = 0.700, 95% CI = 0.502–0.977, P = 0.036).*

As for smoking group, (1) At *COX-1*(*rs5788)*, compared to the CC genotype, the A allele carriers genotype (CA + AA) carriers had a difference between smoking group and control group, this difference remained significant in multivariate analysis by adjusting for age, gender and traditional risk factors *(OR = 1.525, 95% CI = 1.031–2.255, P = 0.034)*. (2) At *COX-2*(*rs3842788*), compared to the GG genotype, the GA genotype and the A allele carriers genotype (GG + GA) had a significant difference between smoking group and control group, and this difference remained significant in multivariate analysis by adjusting for age, gender and traditional risk factors (*OR = 0.631, 95% CI = 0.416–0.957, P = 0.030. OR = 0.626, 95% CI = 0.415–0.943, P = 0.025*).

### MDR analysis

Gene-gene interactions were investigated for ischemic stroke and its subtypes by using MDR, and two models of SVO were statistical significant (*Permutation test P value* < 0.05, Table 4). Among all gene-gene interaction models evaluated, a two-locus model (rs1330344 and rs689466) with the maximum cross-validation consistency (CVC: 10/10) and testing accuracy (0.5304) was regarded as the overall best model in our study. In addition, the best three-locus model (including rs1330344, rs3842788 and rs689466) had a maximum CVC (10) and higher testing accuracy (0.5125).

**Table 4:** MDR models of gene-gene interactions between three SNPs

*SNP*	*IS*					*LAA*				
	Best candidate model	Training accuracy	Testing accuracy	CVC	Permutation test *P* value	Best candidate model	Training accuracy	Testing accuracy	CVC	Permutation test *P* value
1	SNP3	0.5216	0.4981	7 /10	0.206	SNP3	0.5342	0.5342	10/10	0.4270
2	SNP1 SNP3	0.5375	0.5211	10 /10	0.300	SNP1 SNP3	0.5504	0.5346	10/10	0.8400
3	SNP1 SNP2 SNP3	0.5445	0.5176	10 /10	0.330	SNP1 SNP2 SNP3	0.5591	0.5185	10/10	0.5600
*SNP*	*CES*					*SVO*				
	Best candidate model	Training accuracy	Testing accuracy	CVC	Permutation test *P* value	Best candidate model	Training accuracy	Testing accuracy	CVC	Permutation test *P* value
1	SNP1	0.5228	0.4834	5/10	0.7600–0.7610	SNP1	0.53	0.53	10 /10	0.8520
2	SNP1 SNP3	0.5383	0.4776	10/10	0.9510	**SNP1** **SNP3**	0.5428	0.5304	10 /10	**0.0050–0.0060**
3	SNP1 SNP2 SNP3	0.5552	0.4592	10/10	0.7990–0.8000	**SNP1** **SNP2 SNP3**	0.5496	0.5125	10 /10	**0.0190–0.0200**

*P* < 0.05 in bold

COX-1 rs1330344, rs3842788, COX-2 rs689466 signed as SNP1–3

CVC(cross-validation consistency)

### Additive effect analysis

We used multiple logistic regression analysis to analyze the correlation between the additive effect of COX-1 (rs1330344, rs3842788), COX-2 (rs689466) and ischemic stroke. Setting: the risk allele for each locus was 1, and the remainder was 0. We compared the carrying 1, 2, 3 risk loci to carry 0 risk loci without taking into account the specific loci or genes, just carrying any polymorphic loci count. Compared with those carrying none of the variants, individuals carrying one variant, carrying two variants and carrying three variants had no significant differences of the risk of ischemic stroke or its subtypes. (Table 5).

**Table 5:** Additive effect analysis between three SNPs

Risk loci	control(%)	*IS*			*LAA*		
		*n*(%)	OR(95% CI)	*P* value	*n*(%)	OR(95% CI)	*P* value
0	22(2.4)	32(3.0)	Reference		13(3.2)	Reference	
1	230(25.5)	294(27.3)	0.856(0.341–2.151)	0.741	105(25.9)	0.646(0.215–1.942)	0.437
2	586(64.9)	694(64.4)	0.752(0.323–1.752)	0. 509	270(66.7)	0.587 (0.216–1.595)	0.296
3	65(7.2)	58(5.4)	0.400(0.144–1.111)	0.079	17(4.2)	0.231(0.056–0.951)	0.052
Risk loci	control(%)	*CES*			*SVO*		
		*n*(%)	OR(95% CI)	*P* value	*n*(%)	OR(95% CI)	*P* value
0	22(2.4)	4(2.9)	Reference		15(2.8)	Reference	
1	230(25.5)	33(24.3)	0.881(0.231–3.645)	0.861	156(29.1)	1.178(0.388–3.576)	0.773
2	586(64.9)	88(64.7)	0.677 (0.183–2.502)	0.558	336(62.6)	0.747 (0.272–2.050)	0.572
3	65(7.2)	11(8.1)	0.220 (0.023–2.147)	0.193	30(5.6)	0.493 (0.148–1.644)	0.250

Using multivariate analysis after adjusting for age, gender and traditional risk factors

## Discussion

COX gene is closely related to the cause of ischemic stroke. COX-1 gene locates on chromosomes 9q32-q33.3, contains 11 exons and 10introns [31]. COX-1 participates in the normal physiological metabolism of the body and regulates platelet function and blood coagulation [9]. When the vascular endothelial is harmed, it will cause the platelet adhesion, aggregation and activation, leading to platelet consumption. At the same time, the blood system will be activated to megakaryocyte, produce more dense granules, high enzyme activity, active function, and increase aggregation and adhesion ability of large volume platelet, further leading to thrombosis [32]. At present, multiple gene polymorphism loci has been found that in the COX-1 gene coding and non-coding area.

As for COX-1, our results showed that rs1330344 and rs3842788 contributed significantly to the occur of ischemic stroke, but the genotype and allele frequencies at rs5788 had no significant difference between ischemic stroke group and the control group. On one hand, at rs1330344, AA genotype may reduce the susceptibility of ischemic stroke, and CES or SVO. Meanwhile, the A allele carriers also may reduce the susceptibility of ischemic stroke, LAA or CES*.* On the other hand, at rs3842788, the susceptibility of ischemic stroke was fivefold higher in the AA genotype than in the G allele carrier genotype (GG + GA, recessive model); the susceptibility of CES was higher by about 9.8 times; the risk of SVO was higher by about 4.6 times. Our finding matches a study of 859 ischemic stroke patients indicated the rs5788 locus is not related to ischemic stroke in China [23]. But they also suggested that the homozygotes of the minor allele at rs1330344 increase the risk of ischemic stroke recurrence, which was inconsistent with our findings. This difference may be caused we included different type of ischemic stroke patients. Their study included patients with recurrent ischemic stroke, and all of the patients were classified as one type. Instead, our study included first onset of ischemic stroke patients and grouped them by the TOAST classification. In addition, another study of 196 ischemic stroke patients in Korea revealed that the rs3842788 locus is not associated with ischemic stroke, and a study of 469 ischemic stroke patients in USA also got the same results, both of which were inconsistent with our results [24]. This may because these studies all had relatively small samples and involving different ethnic population.

COX-2 gene located on chromosomes 1q25.2-q25.3, contains 10 exons and 9 introns, mainly in the nuclear membrane of cells, so the PGs products can enter the nucleus, which regulates target gene transcription [31]. COX-2 is induced enzyme, exists in vascular endothelial cells, smooth muscle cells and platelets.COX-2 is one of the key enzymes involved in AA generation to PG. [22] It will only highly expressed when the cells affected by various factors participates in physiological and pathological processes of inflammation or tumor [18]. COX-2 expression is 10–20 times higher in patients with atherosclerotic plaques or inflammatory stimuli than in asymptomatic patients with arterial stenosis [33]. Rs20417 and rs689466 are located in the promoter position at COX-2 gene, to participate in regulating gene transcription and specificity, previous studies showed that these loci mutation can change the promoter activity which will affect the expression of COX-2.

As for COX-2, our results showed that the genotype and allele frequencies at rs20417 had no significant difference between the ischemic stroke group and the control group. This conclusion is consistent with a meta-analysis of rs20417 [25] and two studies (411 ischemic stroke patients in China, 469 ischemic patients in USA) [34, 35]. However, there were some controversial researches of rs20417. Francesco et al. [22] observed that the GC and CC genotypes of rs20417 are related to the pathogenesis of ischemic stroke, and C allele may be a genetic protective factor that reduces the incidence of ischemic stroke. Conversely, a study showed that the GC and CC genotypes were commonly found in African–American and European populations affected by ischemic stroke and suggested that COX-2 is an ischemic stroke-susceptible gene [20]. In our study, the difference between ischemic stroke group and control group at rs20417 was not significant, this may due to the difference between our population and genetic heterogeneity. Furthermore, at *COX-2*(*rs689466*), we found the AA genotype carriers had a higher risk of LAA, compared to the G allele carriers genotype (GG + GA). Our finding proved that rs689466 was associated with susceptibility to ischemic stroke in China which matched the results of a Chinese population study (Zhejiang Province-224 patients with LAA and 329 patients with SVO [26]). But the effect of the study was confined to SVO among the stroke subtypes rather than to LAA which was dissimilar to our study. This may be caused by their small amounts of patients. Therefore, it is very important that more large samples and multiethnic studies need to be conducted to verify the association of rs689466 with ischemia stroke in China.

There were some differences observed in genotype and allele frequencies between the diabetes/smoking group and control group, and this difference remained significant in multivariate analysis after adjusting for age, gender and traditional risk factors. Inflammation may play a role in the pathogenesis of type 2 diabetes, COX generates prostaglandins, which negatively modulate glucose-stimulated insulin secretion. This indicated that COX may associate with diabetes [36]. Konheim et al. identified that there was no association of COX2 rs689466 with type 2 diabetes [37]. Their conclusion matched another hospital-based case-control study in Turkey [38]. However, our study found that COX-1 rs1330344 and COX-2 (rs20417, rs689466) were associated with diabetes. The racial difference may cause this inconsistent. Since smoking increases the activity of inflammatory mediators through an increase in leukotriene synthesis essential in atherosclerosis pathogenesis, COX functions as a mediator of the inflammatory response and is also essential in atherosclerosis pathogenesis [39]. Our study found that COX-1 rs5788 and COX-2 rs3842788were associated with smoking, this evidence may explain that the pathogenesis of COX associate with smoking.

We had got some statistically significant positive results from the single gene polymorphism research of COX-1 and COX-2 gene. But these results were not entirely consistent with existing research and cannot fully explain the association between each polymorphism in the same gene. Therefore, we conducted a study on the interaction between multiple loci in ischemic stroke. Our study found that COX-1(rs1330344, rs3842788) and COX-2 rs689466 are associated with SVO. Compared with those patients who do not carry any of the variants, individuals carrying one variant, two variants, and three variants had no significant difference between the ischemic stroke and the control groups. Our previous results showed that rs1330344 reduces the risk of ischemic stroke, including CES and SVO, whereas rs3842788 increases the risk of ischemic stroke, including CES and SVO, rs689466 increases the risk of LAA, and the three loci interact with SVO. Our study also had several limitations. First of all, our study only included the Han population in the southwest of China. Secondly, our study only examined two and three loci of the candidate genes and thus may be unable to cover the entire gene linkage disequilibrium signal. Hence, a linkage disequilibrium map of the gene should be constructed to clarify the relationship between this gene and the pathogenesis of ischemic stroke. Thirdly, this study was limited to two genes and three loci to explore the gene–gene interaction in ischemic stroke. The quantification of the environmental factors of ischemic stroke was relatively difficult. Thus, the role of the interaction of environmental factors was not considered in our study. Therefore, future studies on gene interaction should include environmental and epigenetic factors and should also be performed to enhance our understanding of the genetic structure of ischemic stroke, to investigate the molecular mechanism of ischemic stroke, and to provide a new angle for the diagnosis and treatment of ischemic stroke.

## Conclusion

At rs1330344, AA genotype may reduce the susceptibility of ischemic stroke. At rs3842788, AA genotype may increase the susceptibility of ischemic stroke. At rs689466, AA genotype may increase the susceptibility of large-artery atherosclerosis (LAA). COX-1(rs1330344, rs3842788) and COX-2 rs689466 interacted in small vessel occlusion (SVO), but had no additive effect with ischemic stroke or its subtypes in a Han population from Southwest China.

## Data Availability

The database used and/or analyzed during the current study are available from the corresponding author on reasonable request.

## Abbreviations

CES: Cardioembolic stroke
COX gene: Cyclooxygenase gene
LAA: Large-artery atherosclerosis
SVO: Small vessel occlusion
